# Somatic loss of function mutations in *neurofibromin 1* and *MYC associated factor X* genes identified by exome-wide sequencing in a wild-type GIST case

**DOI:** 10.1186/s12885-015-1872-y

**Published:** 2015-11-10

**Authors:** Martin G. Belinsky, Lori Rink, Kathy Q. Cai, Stephen J. Capuzzi, Yen Hoang, Jeremy Chien, Andrew K. Godwin, Margaret von Mehren

**Affiliations:** Molecular Therapeutics Program, Fox Chase Cancer Center, 333 Cottman Avenue, Philadelphia, PA 19111-2497 USA; Cancer Biology Program, Fox Chase Cancer Center, Philadelphia, PA USA; Division of Chemical Biology and Medicinal Chemistry, University of North Carolina, Chapel Hill, NC USA; Department of Bioinformatics and Biosystems Technology, University of Applied Sciences Wildau, Wildau, Germany; Department of Cancer Biology, University of Kansas Medical Center, Kansas City, KS USA; Department of Pathology and Laboratory Medicine, University of Kansas Medical Center, Kansas City, KS USA

**Keywords:** Gastrointestinal stromal tumor (GIST), Wild type, KIT, *PDGFRA*, Succinate dehydrogenase (*SDH*), *NF1*, *MAX*

## Abstract

**Background:**

Approximately 10–15 % of gastrointestinal stromal tumors (GISTs) lack gain of function mutations in the *KIT* and *platelet-derived growth factor receptor alpha* (*PDGFRA*) genes. An alternate mechanism of oncogenesis through loss of function of the succinate-dehydrogenase (SDH) enzyme complex has been identified for a subset of these “wild type” GISTs.

**Methods:**

Paired tumor and normal DNA from an SDH-intact wild-type GIST case was subjected to whole exome sequencing to identify the pathogenic mechanism(s) in this tumor. Selected findings were further investigated in panels of GIST tumors through Sanger DNA sequencing, quantitative real-time PCR, and immunohistochemical approaches.

**Results:**

A hemizygous frameshift mutation (p.His2261Leufs*4), in the *neurofibromin 1* (*NF1)* gene was identified in the patient’s GIST; however, no germline *NF1* mutation was found. A somatic frameshift mutation (p.Lys54Argfs*31) in the *MYC associated factor X* (*MAX*) gene was also identified. Immunohistochemical analysis for MAX on a large panel of GISTs identified loss of MAX expression in the *MAX*-mutated GIST and in a subset of mainly *KIT*-mutated tumors.

**Conclusion:**

This study suggests that inactivating *NF1* mutations outside the context of neurofibromatosis may be the oncogenic mechanism for a subset of sporadic GIST. In addition, loss of function mutation of the *MAX* gene was identified for the first time in GIST, and a broader role for MAX in GIST progression was suggested.

**Electronic supplementary material:**

The online version of this article (doi:10.1186/s12885-015-1872-y) contains supplementary material, which is available to authorized users.

## Background

Gastrointestinal stromal tumor (GIST) is a mesenchymal neoplasm that originates throughout the GI tract, primarily in the stomach (>50 %) and small intestine (~30 %) [1]. GIST generally presents in older adults, while ~2 % of cases are children [2, 3]. Originally thought to be of smooth muscle origin, immunohistochemical and ultrastructural studies suggest that GIST is related to spindle-shaped cells of the GI tract known as the interstitial cells of Cajal (ICC) [4, 5]. ICC and the majority (95 %) of GIST express the type III receptor tyrosine kinase KIT (CD117), and variably exhibit myoid or neural features. The majority of GISTs exhibit gain of function mutations in KIT or in the related receptor PDGFRA [6, 7]. A subset (~10–15 %) of GISTs in adults lack mutations in the *KIT* and *PDGFRA* genes, as do almost all pediatric cases [8, 9]. The commonly used label of “wild type” (WT) GIST belies the epidemiological, clinico-pathological and molecular heterogeneity that define these tumors. WT GIST occurs in the context of several multitumor syndromes, including the inherited Carney-Stratakis Syndrome (CSS) and the non-familial Carney triad (CT). Manifestations of CSS and CT include gastric GIST and paraganglioma (PGL), a neural crest-derived tumor, while the spectrum of CT neoplasms includes pulmonary chondroma and several other neoplasms [10, 11]. CSS results from loss of function mutations in subunit genes of the succinate-dehydrogenase (SDH) enzyme complex, *SDHB*, *SDHC*, and *SDHD* [12]. Inactivation of the *SDHA* gene subunit has recently been implicated as a causative factor in a subset of apparently sporadic adult WT GIST (reviewed in [13]). GISTs from CT patients do not manifest *SDHX* mutations; however, these tumors are also SDH-deficient, and the molecular underpinning of CT GIST has been attributed to epigenetic silencing of the *SDHC* gene [14]. Pediatric GIST patients share hallmarks of CT-associated GIST, namely early-onset, multi-focal, gastric disease with a predilection towards females [8], and pediatric GIST cases have also been associated with *SDHC* epimutation [15]. Thus the identification of SDH-deficient GIST, also referred to as “type 2”, helps distinguish between *KIT*/*PDGFRA* mutant, or type I GIST, and a majority of wild type GIST.

SDH-intact WT GISTs with alternate oncogenic events have been described. Mutations in the serine-threonine kinase gene *BRAF* have recently been identified in approximately 5–15 % of sporadic wild type GIST [16]. These tumors are generally KIT-positive with spindle cell or mixed morphology, and are found primarily in the small intestine in adult cases. Approximately 1–2 % of GISTs occur in the context of neurofibromatosis type I (NF1) [1], an autosomal dominant disorder with skin and ophthalmologic manifestations that predisposes to a variety of benign and malignant tumors. GIST in NF1 individuals also present typically in the small bowel with spindle-cell morphology, are found in men and women at a younger median age than *KIT*/*PDGFRA* mutant GIST, and are often multifocal [17, 18]. Neurofibromatosis is due to germline mutations in neurofibromin 1, a RAS-GAP protein and negative regulator for RAS signaling, and germline *NF1* mutations accompanied by somatic events have been identified in NF1 GIST cases [19].

In this report we describe whole exome sequencing (WES) of a particularly complex, SDH-intact wild type GIST case. The WES analysis identified for the first time the somatic inactivation of *NF1* in a GIST outside the context of NF1 syndrome. A novel somatic loss of function mutation in the *MYC-associated factor X* (*MAX*) gene was also identified. Immunohistochemical studies of a panel of GISTs identified deficiencies in MAX expression in a number of tumors. Implications for these and other identified mutations are discussed.

## Methods

### Preparation of genomic DNA and total RNA

De-identified tumor samples and normal blood were obtained following written informed consent from the Fox Chase Cancer Center Biosample Repository. The protocol was approved by the Fox Chase Cancer Center Institutional Review Board (#03-848). The isolation and characterization of genomic DNA and total RNA from frozen tumor specimens embedded in optimum-temperature cutting medium has been described [20].

### Whole exome sequencing data analysis

Exome-enriched genomic libraries (TruSeq, Illumina, San Diaego CA) from normal and tumor DNA were subjected to paired-end 100 bp sequencing on the Illumina HiSeq 2000 instrument. Reads were mapped to the 1000Genome Project reference human genome (Hg19 corresponding v37) using the BWA aligner [21] and mapped reads were sorted, merged, and de-duplicated (Picard), yielding an average of 51.6 million unique mapped reads per sample. GATK realignment was used to realign reads locally in areas surrounding insertions and deletions (indels) [22, 23]. Variant calling and filtering was performed using GATK UnifiedGenotyper [22, 23] and single nucleotide variants (SNVs) annotated with modified ANNOVAR [24]. This pipeline yielded an average SNV rate of ~ 0.34 % per sample. The downstream analysis of SNVs and indels was done by custom Perl scripts. Non-synonymous, potentially deleterious coding region variants, splice-site mutations, and indels that were predicted to be present in the tumor only, were visually confirmed on the Integrative Genomics Viewer (IGV) [25], and confirmed by exon-based Sanger sequencing. Confirmed somatic indels, and deleterious missense mutations predicted by the SIFT algorithm [26] and confirmed by a consensus approach [27] are listed in Table 1. Mutation nomenclature conforms to the recommendations of the Human Genome Variation Society [28].

**Table 1:** Confirmed somatic mutations

Gene symbol	UniProt accession^a^	Genomic coordinate^b^	Exon	Mutation (cDNA)	Mutation (protein)	Allele frequency	Consensus effect^c^
*NF1*	P21359	chr17:29665119	44	c.6781_6782insTT	p.His2240Leufs*4	100	n/a^d^
*MAX*	P61244	chr14:65560437	3	c.160delC	p.Gln54Lysfs*10	91	n/a^d^
*RTN4*	Q9NQC3	chr2:55200745	8	c.3486_3490delAGAT	p.Asp1163Ilefs2	36	n/a^d^
*CCDC66*	A2RUB6	chr3:56650054	13	c.1818_1819insCCT	p.Ser606_Lys607insPro	29	n/a^d^
*MVD*	P53602	chr16:88725087	2	c.112T>A	p.S38T	58	Deleterious
*MAFA*	Q8NHW3	chr8:144511807	1	c.770A>T	p.Q257L	56	Likely deleterious
*RNF123*	Q5XPI4	chr3:49751544	31	c.2947T>G	p.Y983D	52	Likely deleterious
*SPIN4*	Q56A73	chrX:62570610	1	c.89G>T	p.R30L	47	Likely deleterious
*SELP*	P16109	chr1:169565261	12	c.2003G>T	p.C668F	49	Likely deleterious

^a^ http://www.uniprot.org; ^b^Hg19; ^c^ http://www.mypeg.info; ^d^Not applicable

### Sanger sequencing

Primers for amplification and sequencing of *KIT* (exons 9, 11, 13, 17), *PDGFRA* (exons 12, 14, 18), and *BRAF* (exons 11,15) have been described [29], as have primers for *SDHA* [30] and *SDHB-D* [31]. Primer sequences for confirmation of mutations listed in Table 1 and *MAX* genomic sequencing are shown in Additional file 1: Table S1. Relevant exons were PCR-amplified from genomic DNA and subjected to Sanger sequencing (Beckman Coulter Genomics).

### Immunohistochemical analysis

GIST tissue microarrays (TMAs) were constructed in conjunction with the FCCC Biosample Repository. H&E-stained sections from paraffin-embedded tissue blocks were evaluated by a pathologist for tumor content and cellularity, and two cores from each block were selected for the TMA. Each TMA consists of ~ 30 GIST specimens along with normal tissue sections. IHC for MAX was performed with the SC-197 antibody (Santa Cruz Biotechnology, Dallas TX) at a 1:400 dilution with antigen retrieval. Aperio Digital Pathology (Leica Biosystems, Buffalo Grove, IL) was used to capture and quantify MAX-stained TMAs using the nuclear algorithm. MAX-deficient cases were confirmed on whole-tissue sections, as were a subset of MAX-positive cases. IHC for KIT was performed as described [32].

### Gene expression analysis

Random-primed cDNA was prepared from 2 μg total RNA using the High Capacity cDNA Reverse Transcription KIT (Life Technologies). RNA expression was measured by real-time PCR (qRT-PCR) on an ABI PRISM 7900 HT Sequence Detection System using fluorescein phosphoramidite (FAM) primer/probe sets (Applied Biosystems). RNA expression data for MAX were normalized using hypoxanthine guanine phosphoribosyl transferase 1 (HPRT1) and glucuronidase beta (GUSB). Taqmen sets used were Hs99999909_m1 (HPRT1), Hs99999908_m1 (GUSB), and Hs00811069_g1 (MAX).

## Results

The patient first presented at the age of 54 with a high-risk GIST of the small bowel. Resections of several local and distant recurrences were documented over several years, and the patient was treated with imatinib, sunitinib, and several additional targeted agents. A locally recurrent 1.5 cm small bowel tumor was resected, and a small portion of flash-frozen tumor and a whole blood sample were provided following informed consent. Formalin-fixed paraffin embedded (FFPE) tissue from an earlier small bowel resection was also available. Histologic evaluation of these specimens indicated a high-cellularity spindle-cell tumor with a high mitotic index (5-10/50 HPF) and no necrosis.

Sanger sequencing of DNA from the flash-frozen tumor indicated that no mutations were present in the “hotspot” exons of *KIT*, *PDGFRA*, or *BRAF*. Exon-based sequencing of the SDH complex subunit genes *SDHA-D* identified no *SDHX* mutations, and the tumor was immunohistochemically positive for SDHB expression [20]. These analyses suggested that the case belonged to the small subset of SDH-intact and *KIT*, *PDGFA*, *BRAF* wild-type GIST, for which no clear molecular pathogenic mechanism has been established. DNA from this GIST and from the patient’s blood was therefore subjected to whole-exome sequencing (WES) (see Materials and Methods).

**Fig. 1:**
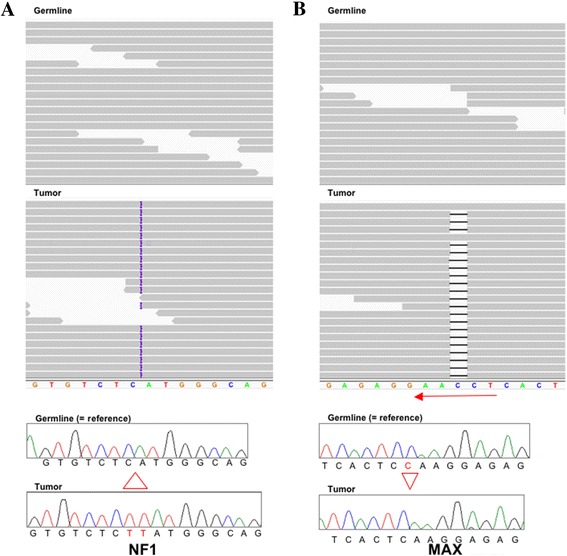
**a** WES (top) and Sanger (bottom) sequencing showing the two-base (TT) insertion in *NF1*. A subset of reads visualized on the Integrative Genomics Viewer (IGV) shows the insertion represented by the purple bar in 100 % of the tumor reads, as confirmed by the chromatogram below. **b** Top and bottom panels show the single-base (**c**) deletion in the *MAX* gene in a majority (~90 %) of reads, again confirmed by the chromatogram below. Red arrow indicates direction of transcription for *MAX*

WES analysis identified a two-base insertion (c.6781_6782insTT; p.His2240Leufs*4) in exon 44 in the *Neurofibromatosis type I* gene (*NF1*) that was confirmed by Sanger sequencing (Fig. 1a and Table 1). The mutation is not seen in the patient’s germline DNA, and the wild type allele is not represented in the tumor in either the WES or Sanger analysis. A previously reported single-nucleotide polymorphism (SNP) array analysis of this GIST (case 26 [29]) identified copy-number loss of the region encompassing the *NF1* gene locus, suggesting somatic *NF1* gene inactivation through the frameshift mutation combined with loss of the wild type gene. This particular *NF1* mutation was not found in the COSMIC [33], ClinVar [34] or Leiden Open Variation databases [35]. To our knowledge this is the first reported example of GIST with an inactivating *NF1* mutation outside the context of the NF1 syndrome. This sporadic GIST does share certain characteristics with GISTs from NF1 patients, such as small bowel origin, spindled-cell morphology, and immunopositivity for KIT and SDHB [36]. It is reasonable to suggest that somatic *NF1* gene inactivation may be a causative factor in formation of the patient’s disease.

**Fig. 2:**
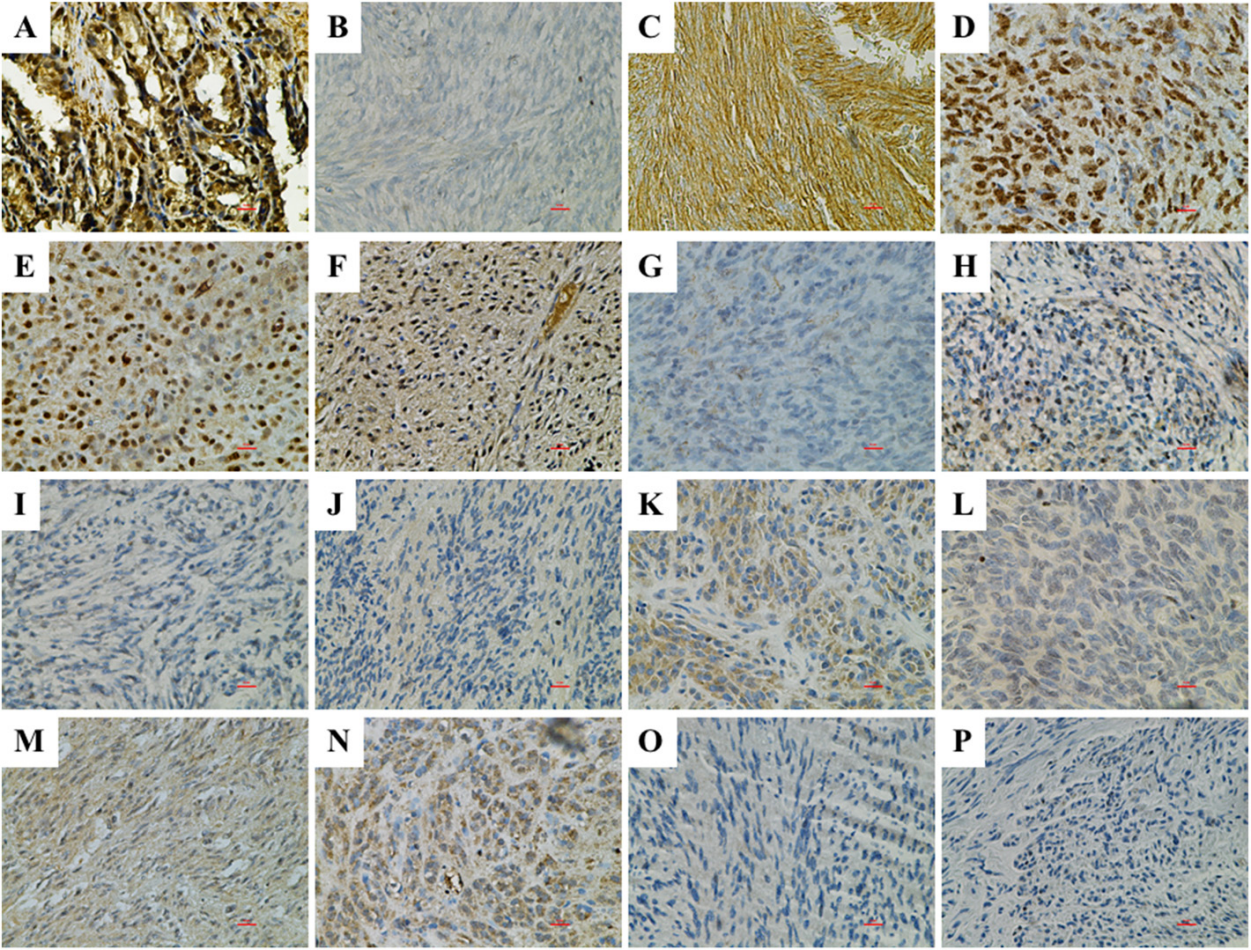
Immunohistochemistry for MAX and KIT. **a** Control staining of nuclear MAX in seminal vesicles. **b** Negative staining for MAX in index case. **c** Strong plasma membrane straining for KIT/CD117 in index case. **d**-**f** Strong nuclear staining for MAX in MAX-positive GISTs. **g**-**p** Mainly negative nuclear staining for MAX in GIST cases 1–10. Red bar: 10 μM

WES analysis also identified a truncating frameshift mutation (c.160delC; p.Gln54Lysfs*10) in exon 3 of the *MYC-associated factor* (*MAX*) gene in the tumor DNA (Fig. 1b and Table 1). This mutation was found in 91 % of the WES reads. MAX is a basic helix-loop-helix (H-L-H) leucine zipper (LZ) transcription factor and a key member of the MYC/MAX/MXD network [37]. This truncating mutation is predicted to disrupt domains responsible for MAX homo-dimerization and hetero-dimerization [38]. Inactivating *MAX* mutations have recently been reported in inherited and sporadic PGL and pheochromocytomas (PCC) [39, 40], and in small cell lung cancer (SCLC) specimens [41]. No mutations in *MAX* have been reported in GIST, and we did not find additional *MAX* mutations in a sample set of 16 wild-type tumors from 11 patients. However, these WT GIST are all SDH-negative tumors, and exhibit other characteristics (e.g. gastric location, epithelioid cell morphology, lack of genome complexity) [20] that are not found in our index case. An earlier report documented a significant reduction in MAX expression in association with copy-number loss surrounding the *MAX* gene locus in a set of kinase-mutant GISTs [42]. We hypothesized that reduction or loss of MAX expression may be associated with mutant GIST. To test this hypothesis, immunohistochemistry from MAX was carried out on a series of ~80 GIST specimens contained on 3 GIST tissue microarrays (TMAs). The antibody was first tested against human seminal vesicle tissue, which exhibited strong nuclear staining (Fig. 2, panel a). Staining of the GIST from the patient’s earlier resection confirmed complete absence of MAX and strong plasma membrane staining for KIT in the tumor (Fig. 2, panels b and c respectively). IHC analysis of the 3 TMAs identified a wide range of MAX staining in GIST sections, ranging from strong, widely distributed nuclear staining to complete or near-complete absence of staining. Images were captured and quantified (Materials and Methods). Nuclear staining intensity (0–3) and distribution (0–100) were combined to generate nuclear H-Scores, with a potential range of 0–300. The mean nuclear H-score across the GIST samples on the TMA was 99.7 (0–252). Visual re-examination of the stained spots suggested H-scores <20 as a reasonable cutoff below which very little positive nuclear staining was seen. Whole tissue sections from a number of these tumors were re-stained with MAX, and the TMA results confirmed in 10 of these cases (Table 2 and Fig. 2). Several GISTs that exhibited strong or intermediate nuclear MAX staining are shown in Fig. 2 (panels d-f, with H-scores of 210, 192, and 84.8, respectively). In Fig. 2, panels g-p correspond to Cases 1–10 in Table 2. These images generally corroborate the low nuclear MAX H-scores from the TMA, although some tissues show isolated nuclear staining (e.g. panel h).

**Table 2:** Description of MAX-negative cases

Case	H-score^a^	Age/Sex	Genotype	Risk^b^	Site
1	0.0	71/M	*PDGRA* exon 18	H	Gastric
2	3.2	48/F	*KIT* exon 11	H	Gastric
3	5.0	83/M	*KIT* exon 11	I	Gastric
4	5.9	39/M	*KIT* exon 11	L	Gastric
5	7.4	35/M	*KIT* exon 11	H	Other
6	10.9	n/a^c^	*KIT* exon 11	H	n/a^c^
7	13.6	46/M	*KIT* exon 11	I	Gastric
8	13.9	68/F	*KIT* exon 11, 17	H	Small bowel
9	14.2	72/M	*KIT* exon 11	I	Gastric
10	17.6	50/M	*KIT* exon 11	I	Gastric

^a^H-score: nuclear staining intensity x percentage; ^b^GIST prognosis based on tumor site, size, mitotic index. H= high, I = intermediate, L = low; ^c^Not available

Table 2 lists the clinico-pathological characteristics of the 10 MAX-deficient cases. Nine cases harbor *KIT* exon 11 mutations, while one patient’s tumor exhibited a mutation in exon 18 of *PDGFRA*. The cases are mainly males (7 of 9 available), with an average age of presentation of 56.9 years. Most were of gastric origin, and nine were high- or intermediate-risk GIST. None of these parameters varied significantly however when compared to the rest of the sample set (Additional file 2: Table S2). Exon-based Sanger sequencing of eight of these cases did not identify any mutations in *MAX*.

Table 1 lists several other confirmed somatic mutations in our index case: two indels and five missense mutations that were predicted to affect protein structure or function. The variant calls represent ~30–60 % of the total reads at these positions, and exon-based Sanger sequencing confirmed that these mutations were at most heterozygous in the tumors. A frameshift deletion in *reticulon 4 (RTN4*) was identified, along with an in-frame insertion in the coiled-coiled domain-containing protein *CCDC66*. Missense mutations were identified in 5 genes: the gene encoding the enzyme mevalonate decarboxylase (*MVD*) that catalyzes an early step in cholesterol synthesis; *MAFA* (*musculoaponeurotic fibrosarcoma oncogene family, protein A*), a transcription factor that controls insulin gene expression in the pancreas; the ring-finger protein gene *RNF123,* which acts as a ubiquitin ligase towards the cyclin-dependent kinase inhibitor KIP1; a member of the spindlin family of chromatin readers (*SPIN4*); and the *SELP (*selectin P) gene, encoding a calcium-dependent receptor that mediates the interaction of activated endothelial cells or platelets with leukocytes.

## Discussion

This study is the first report of somatic inactivation of the *NF1* gene in a sporadic SDH-intact GIST lacking gain of function mutations in *KIT*, *PDGFR*, and *BRAF*. Although tissue from the primary tumor from this patient was not available for analysis, it is reasonable to suggest that somatic inactivation of *NF1* may have been an early causative event in this case. NF1 patients are ~45X more likely to develop GIST than the general population [17], and it has been estimated that 1–2 % of GIST arise in patients with NF1 [1]. GISTs in NF1 patients commonly lack activating mutations in the KIT and PDGFRA receptors [17, 18, 43], and may owe their incidence to germline *NF1* mutation coupled with somatic second hits, as has been demonstrated in some cases [19, 44, 45]. It is perhaps not surprising to find *NF1* gene inactivation in a sporadic wild-type GIST, as *NF1* somatic mutations have been identified in a number of non-NF1-associated tumor types (reviewed in [44]). Moreover, while PCCs are known to occur in the context of NF1 [46], *NF1* somatic mutations were also identified in a high percentage (21/61) of sporadic PCC selected for specific gene expression patterns or low levels of *NF1* gene expression [47]. The finding of *NF1* gene inactivation in sporadic GIST has diagnostic implications, as the molecular identification of mutations in the large and complex *NF1* gene is a challenging task. A comprehensive approach combining NF1 transcript and genomic sequencing with multiplex ligation dependent probe amplification and other techniques for detection of gene duplications and deletions has been used to detect mutations in up to 95% of NF1 cases [48]. Immunohistochemical approaches using available anti-NF1 antibodies have been largely unsuccessful in identifying NF1-mutated PCC with a high degree of sensitivity or specificity [47, 49]. Recently, WES approaches have been used to identify the germline and somatic *NF1* events in various tumors from an NF1 patient [50]. Whether *NF1* inactivation is a common event in sporadic, SDH-intact wild type GIST is an open question. In a recent transcriptome-sequencing study [51], no *NF1* mutations were identified in two SDH intact wild-type GIST, although it has been shown that only a portion of exonic variants are typically captured by RNA-seq approaches [52]. Interestingly, next-generation sequencing of eight SDH-negative GIST cases using a targeted cancer-associated gene capture library identified a low-frequency (8 %) frameshift *NF1* mutation in a GIST that also harbored an activating *KRAS* gene mutation (G12V) [53].

The identification of a loss of function mutation in the *MAX* gene is a novel finding in GIST. As a hetero-dimeric partner for MYC, MAX was originally thought to be required for oncogenic pathways initiated by MYC over-expression [54]. However, in MAX-deficient rat PC12 PCC cell lines it has been shown that MAX is dispensable for MYC transcriptional regulation [55]. Intriguingly, MAX inactivation has recently been implicated in both inherited and sporadic PCC and PGL cases [39, 40]. Mutation of *MAX* is a relatively rare event in these tumors, accounting for 1.12 % of hereditary PCC/PGL lacking mutations in other susceptibility genes and 1.65 % of sporadic cases. In the familial PCC cases preferential transmission of the disease from the paternal allele was observed, along with a tendency towards aggressive behavior. A recent report also describes MAX inactivation in 6 % of primary SCLC specimens [41]. The authors suggest that SCLC, like PCC, may arise from neuroendocrine cells or differentiate towards neural features, which may explain the shared mechanism of MAX-associated oncogenesis. Similarly, sub-populations of GISTs may also variably exhibit neural properties or markers, and in a recent report a set of SDH-intact WT GIST was shown to exhibit high relative expression of neural markers, along with expression of members of the insulin-like growth family network [56]. In SCLC, inactivating *MAX* mutations were found to be mutually exclusive with amplifications of hetero-dimeric partners *MYC*, *MYCL1*, and *MYCN*, and mutations in *BRG1*, which encodes an ATPase of the SWI/SNF chromatin -remodeling complex that regulates expression of MYC, MYC target genes, as well as MAX. In GIST, the contributions of the MYC/MAX/MXD network to pathogenesis have not been extensively described. There have been descriptions of amplification of the *MYC* gene locus on chromosome 8q [57, 58], and reduced mRNA expression of the *MAX* gene associated with copy number loss of chromosome14q [42]. These secondary chromosome aberrations are common in *KIT*/*PDGFRA-*mutated GIST [59]. In this report we used immunohistochemical approaches to identify reduced/absent MAX nuclear staining in 10/78 (~13 %) of GIST cases analyzed, in addition to the index case. We found no additional *MAX* mutations in these tumors, and *MAX* RNA expression was only marginally and not significantly reduced (1.3-fold, *P* = 0.47) compared to the remaining MAX-positive cases. Further investigations into the mechanism(s) of MAX dysregulation and its contribution to pathogenesis in GIST are warranted.

In addition to inactivating *NF1* and *MAX* mutations found in our index case, we also identified a heterozygous 4-base deletion in *RTN4* and an in-frame insertion in *CCD66*, as well as potentially pathogenic substitutions in the *MVD*, *MAFA*, *RNF123*, *SPIN4*, and *SELP* genes. Although to our knowledge these genes have not been studied in GIST, a recent systems biological approach to identifying key transcriptional regulators in GIST and leiomyosarcoma identified nine differentially expressed genes, including the *MYC* gene, the *MAF* gene which encodes another basic leucine zipper (bZIP)-containing transcription factor closely related to *MAFA*, and another coiled-coil domain containing transcription factor gene, *CCDC6* [60]. The MAF proteins are members of the AP1 family: the large MAF proteins contain transactivation domains and are considered onco-proteins by virtue of their ability to transform primary cells and induce tumors in various animal models (reviewed in [61]). Interestingly, the Q257L substitution we identified is located in the MAFA bZIP domain, very close to the predicted DNA-binding domain that has been shown to be required for MAFA transformation activity in avian fibroblasts [62]. The substitution we identified may affect the specificity or avidity of MAFA binding to its target sequences.

## Conclusions

In conclusion, next-generation sequencing of an SDH-intact, *KIT*, *PDGFR*, *BRAF* wild type GIST identified for the first time somatic loss of function mutations in two tumor-suppressor genes, *NF1* and *MAX*. Somatic inactivation of neurofibromin should be explored as a potential oncogenic mechanism in this subset of GIST. The identification of *MAX* inactivation provides another etiological link between GIST and PGL/PCC, in addition to mutations of the *NF1* gene and mutations in the subunit genes of the SDH complex that have been identified in these tumors.

## Availability of supporting data

The data supporting the results of this article are included within the article and its additional files.

## Additional files

Additional file 1: Table S1. Primers for Sanger sequencing. Nucleotide sequences are listed for primers used for exon-based validation of somatic mutations listed in Table 1, and for all exons of the *MAX* gene. (DOC 42 kb) Additional file 2: Table S2. Characteristics of MAX-negative and MAX-positive GIST cases. Selected molecular, demographic, and clinical characteristics of GIST sample sets stratified by MAX immunohistochemical expression. (DOC 60 kb)

## Abbreviations

CSS: Carney-Stratakis syndrome
CT: Carney triad
GIST: gastrointestinal stromal tumors
H&E: hematoxylin and eosin
ICC: interstitial cells of Cajal
IHC: immunohistochemistry
Indels: insertions and deletions
MAX: Myc-associated factor X
NF1: neurofibromatosis type 1
PDGFRA: platelet-derived growth factor receptor alpha
PGL: paraganglioma
PCC: pheochromocytoma
qRT-PCR: quantitative real-time polymerase chain reaction
SCLC: small-cell lung cancer
SDH: succinate dehydrogenase
SNV: single nucleotide variant
SNP: single nucleotide polymorphism
TMA: tissue microarray
WES: whole exome sequencing
