# Supplementary material for: Somatic loss of function mutations in neurofibromin 1 and MYC associated factor X genes identified by exome-wide sequencing in a wild-type GIST case
Source: BMC Cancer. 2015 Nov 10;15:887. doi: 10.1186/s12885-015-1872-y (PMC4641358; doi:10.1186/s12885-015-1872-y)
Supplement: Additional file 1: Table S1. — Primers for Sanger sequencing. Nucleotide sequences are listed for primers used for exon-based validation of somatic mutations listed in Table 1, and for all exons of the MAX gene. (DOC 42 kb) [file 12885_2015_1872_MOESM1_ESM.doc]

| Supplementary Table 1. Primers for Sanger sequencing. | |
| --- | --- |
| Gene exon-orientation | Sequence (5’-> 3’) |
| *MAX* 1-F | GTTGTTGTCGGTGACTTCCC |
| *MAX* 1-R | CCCAACCTCCAGTCCCAG |
| *MAX* 2-F | ACCCTCCGCTTCCTCTGC |
| *MAX* 2-R | TCCACTTACATAGTGGCATCTGG |
| *MAX* 3-F | CTCGAGAAATCCTTCCCAGTC |
| *MAX* 3-R | TCCCAATAGGTGAGTGCTCTG |
| *MAX* 4-F | CCCACCTTACCCTCTCGTTTC |
| *MAX* 4-R | CCATGACTGGCTCTGACTCTG |
| *MAX* 5-F | AGAACAGGCTGGACCCTGAG |
| *MAX* 5-R | GTTCTGAGGGCTCTACCAACG |
| *NF1* 44-F | TTCTGTGGATCTTTTAATTGCAG |
| *NF1* 44-R | TGAGAACCATAAATATTTGGGAGA |
| *RTN4* 6-F | TTCTTAAGCCTTTAGCTTGACACA |
| *RTN4* 6-R | TTCTCACAATCCAGCACACC |
| *CCDC66* 13-F | CAAATGGAATATAATGCATCTAACA |
| *CCDC66* 13-R | AAACCAAACCACAATTTCCA |
| *MVD* 2-F | GGTGAGAGACGGGACTGAGG |
| *MVD* 2-R | GGAGACCACAGTGAACCATGA |
| *MAFA* 1-F | CAGCTGGTGTCCATGTCG |
| *MAFA* 1-R | TTGTACAGGTCCCGCTCTTT |
| *RNF123* 31-F | TGGAGGGTAAGCCTGACTCG |
| *RNF123* 31-R | GTCCCAGACTCCCTACGTGTC |
| *SPIN4* 1-F | AAGCCCTCTTAATCCCTGAGC |
| *SPIN4* 1-R | CCAAGGATGAATGGAAGGGTA |
| *SELP* 12-F | CAGGAGCCTCCCTTGTTATG |
| *SELP* 12-R | CCATTGTGCCTGTATGTTGAA |
