# Supplementary material for: Somatic loss of function mutations in neurofibromin 1 and MYC associated factor X genes identified by exome-wide sequencing in a wild-type GIST case
Source: BMC Cancer. 2015 Nov 10;15:887. doi: 10.1186/s12885-015-1872-y (PMC4641358; doi:10.1186/s12885-015-1872-y)
Supplement: Additional file 2: Table S2. — Characteristics of MAX-negative and MAX-positive GIST cases. Selected molecular, demographic, and clinical characteristics of GIST sample sets stratified by MAX immunohistochemical expression. (DOC 60 kb) [file 12885_2015_1872_MOESM2_ESM.doc]

| Supplementary Table 2. Characteristics of MAX-negative^a^ and MAX-positive GIST cases. | | | |
| --- | --- | --- | --- |
|  | MAX-negative (n=10) | MAX-positive (n=68) | *P* value |
|  |  |  |  |
| Genotype |  |  | 0.527^b^ |
| *KIT*-mutant | 9 | 46 |  |
| *PDGFRA*-mutant | 1 | 10 |  |
| Wild type | 0 | 5 |  |
| n/a^b^ | 0 | 7 |  |
|  |  |  |  |
| Gender |  |  | 0.191^b^ |
| Male | 7 | 30 |  |
| Female | 2 | 25 |  |
| n/a^d^ | 1 | 13 |  |
|  |  |  |  |
| Age (mean ± SD) | 56.9 ± 16.9 | 59.3 ± 13.9 | 0.638^c^ |
|  |  |  |  |
| Primary tumor site |  |  | 0.354^b^ |
| Stomach | 7 | 29 |  |
| Small bowel | 1 | 17 |  |
| Other | 1 | 9 |  |
| n/a^b^ | 1 | 13 |  |
|  |  |  |  |
| Risk |  |  | 0.413 ^b^ |
| High | 5 | 17 |  |
| Intermediate | 4 | 7 |  |
| Low | 1 | 8 |  |
| n/a^b^ | 0 | 36 |  |
| ^a^Aperio H-scores <20 were considered negative for MAX IHC.; ^b^Chi-square test; ^c^Student’s T-test; ^d^Not available | | | |
